# Supplementary material for: Complex‐centric proteome profiling by SEC‐SWATH‐MS
Source: Mol Syst Biol. 2019 Jan 14;15(1):e8438. doi: 10.15252/msb.20188438 (PMC6346213; doi:10.15252/msb.20188438)
Supplement: Supplementary file 7 — Dataset EV6 [file MSB-15-e8438-s007.zip › feature_plots_bioplex/Q13601.pdf]

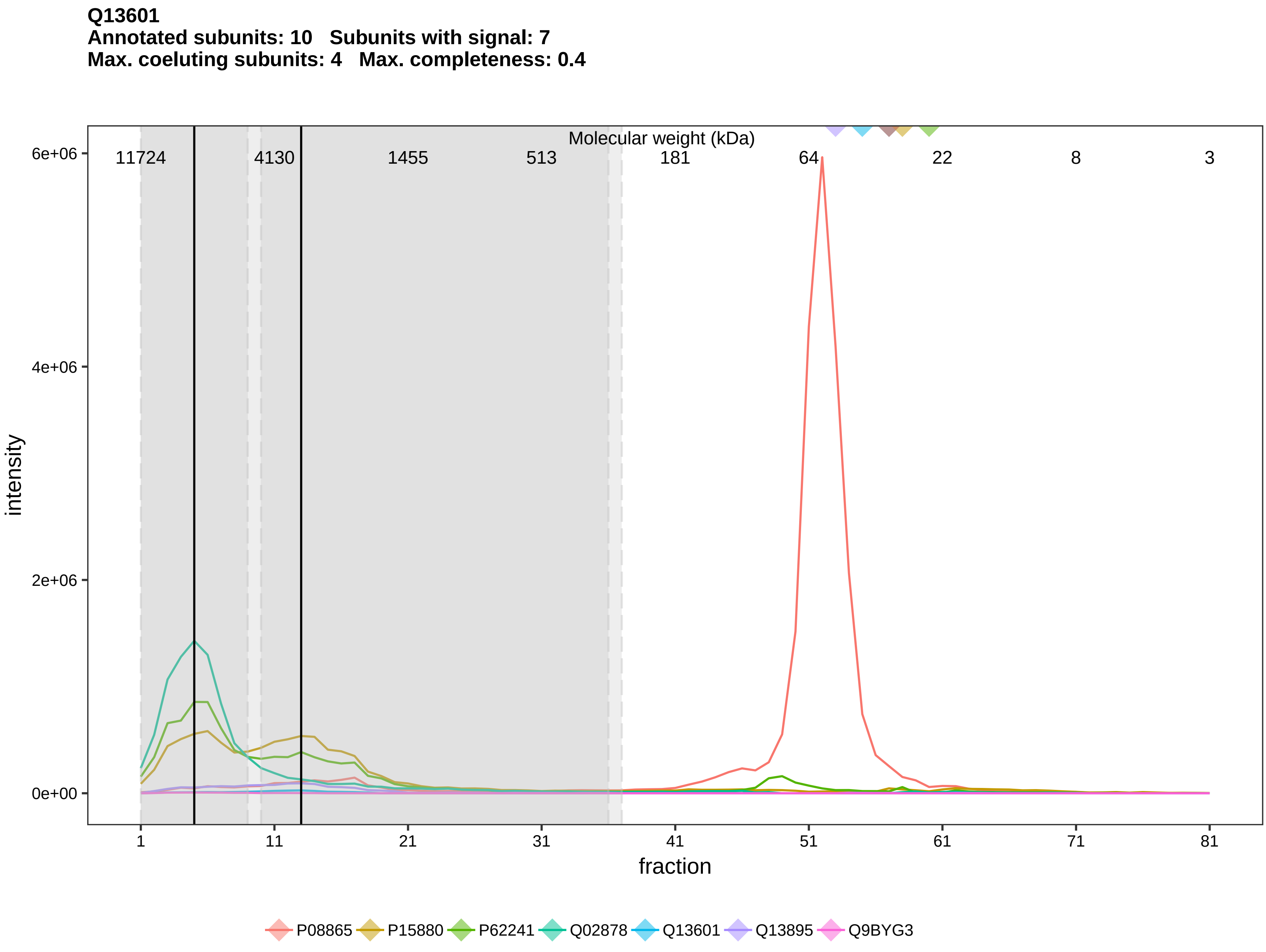

Q13601
Annotated subunits: 10   Subunits with signal: 7
Max. coeluting subunits: 4   Max. completeness: 0.4
Molecular weight (kDa)
11724
4130
1455
513
181
64
22
8
3
6e+06
4e+06
2e+06
0e+00
intensity
1
11
21
31
41
51
61
71
81
fraction
P08865
P15880
P62241
Q02878
Q13601
Q13895
Q9BYG3
